# Mitochondrial genome of *Diachrysia nadeja* (Lepidoptera: Noctuoidea: Noctuidae) and phylogenetic analysis

**DOI:** 10.1080/23802359.2020.1870881

**Published:** 2021-02-11

**Authors:** Shanshan Gao, Shuang Xue, Yuanchen Zhang, Jingshun Wang, Kunpeng Zhang

**Affiliations:** a College of Biology and Food Engineering, Anyang Institute of Technology, Anyang, China; b College of Plant Protection, Henan Agricultural University, Zhengzhou, China

**Keywords:** Noctuidae, mitochondrial genome, *Diachrysia nadeja*, phylogenetic analysis

## Abstract

*Diachrysia nadeja* is a polyphagous herbivorous moth within the family Noctuidae. In this study, we sequenced and analyzed the complete mitochondrial genome (mitogenome) of *D. nadeja*. This mitogenome was 15,242 bp long and encoded 13 protein-coding genes (PCGs), 22 transfer RNA genes (tRNAs) and 2 ribosomal RNA unit genes (rRNAs). Gene order was conserved and identical to most other previously sequenced Noctuidae. Except for *cox1* started with CGA, all other PCGs started with the standard ATN codons. Most of the PCGs terminated with the stop codon TAA, whereas *cox1*, *cox2,* and *nad4* end with the incomplete codon T−. The whole mitogenome exhibited heavy AT nucleotide bias (80.5%). Phylogenetic analysis showed that *D. nadeja* got together with three *Ctenoplusia* species (*C. agnata*, *C. limbirena,* and *C. albostriata*) with high support value, indicating *Diachrysia* had a closer relationship with *Ctenoplusia* within Noctuidae.

Plusiinae (Lepidoptera: Noctuidae) is a main large subfamily of worldwide distribution, with more than 500 species worldwide, and they are spread from the tropics to the arctic (Ronkay et al. 2008). Species of Plusiinae can be easily distinguished from other subfamilies of Noctuidae by the following characters: golden spots or silvery stigma on the forewing, lashed eyes, and large scale tufts on the thorax, and the larvae bearing two pairs of prolegs on abdominal segments V and VI (Ronkay et al. 2008; Behounek et al. 2010). *Diachrysia nadeja* (Oberthür, 1880), one of the species in Plusiinae, has recorded in China, Euro-Siberian and Japan (Goater et al. 2003). *Diachrysia nadeja* is a polyphagous herbivorous moth, with the larvae feed on plants of Chenopodiaceae, Polygonaceae, Urticaceae, Fabaceae, Rubiaceae, Plantaginaceae, Lamiaceae, and Asteraceae (Leley 2016).

Specimens of *D. nadeja* were collected from Dalian City, Liaoning Province, China (38°90′N, 121°45′E, August 2019) and were stored in Entomological Museum of Anyang Institute of Technology (Accession number AIT-E-DIA02). Total genomic DNA was extracted from tissues using DNeasy DNA Extraction kit (Qiagen, Hilden, Germany). The mitogenome sequence of *D. nadeja* was generated using Illumina HiSeq 2500 Sequencing System (Illumina, San Diego, CA, USA). In total, 5.6 G raw reads were obtained, quality-trimmed, and assembled using MITObim v 1.7 (Hahn et al. 2013). By comparison with the homologous sequences of other Noctuidae species from GenBank, the mitogenome of *D. nadeja* was annotated using software GENEIOUS R8 (Biomatters Ltd., Auckland, New Zealand).

The complete mitogenome of *D. nadeja* is 15,242 bp in length (GenBank accession no. MT916722), and containing the typical set of 13 protein-coding, 2 rRNA, and 22 tRNA genes, and one non-coding AT-rich region. Gene order was conserved and identical to most other previously sequenced Noctuidae (Timmermans et al. 2014; Li et al. 2015; Yao et al. 2018; Huang et al. 2019). The overall base composition of the mitogenome was estimated to be A 39.1%, T 41.4%, C 11.6% and G 7.9%, with a high A + T content of 80.5%. Except for *cox1* started with CGA, all other PCGs started with the standard ATN codons (seven ATG, two ATT, two ATA and one ATC). Most of the PCGs terminated with the stop codon TAA, whereas *cox1*, *cox2,* and *nad4* end with the incomplete codon T−. The 22 tRNA genes vary from 63 bp (*trnR*) to 71 bp (*trnK*). Two rRNA genes (*rrnL* and *rrnS*) locate at *trnL1*/*trnV* and *trnV*/control region, respectively. The lengths of *rrnL* and *rrnS* in *M. calida* are 1346 and 785 bp, with the AT contents of 84.4 and 85.3%, respectively.

Phylogenetic analysis was performed based on the nucleotide sequences of 13 PCGs from 22 Lepidoptera species. Phylogenetic tree was constructed through raxmlGUI 1.5 (Silvestro and Michalak 2012) (Figure 1). Results showed that the new sequenced species *D. nadeja* got together with three *Ctenoplusia* species (*C. agnata*, *C. limbirena,* and *C. albostriata*) with high support value (BS = 100), indicating *Diachrysia* has a closer relationship with *Ctenoplusia* within Noctuidae. In conclusion, the mitogenome of *D. nadeja* is sequenced in this study and can provide essential DNA molecular data for further phylogenetic and evolutionary analysis of Noctuidae.

**Figure 1.**
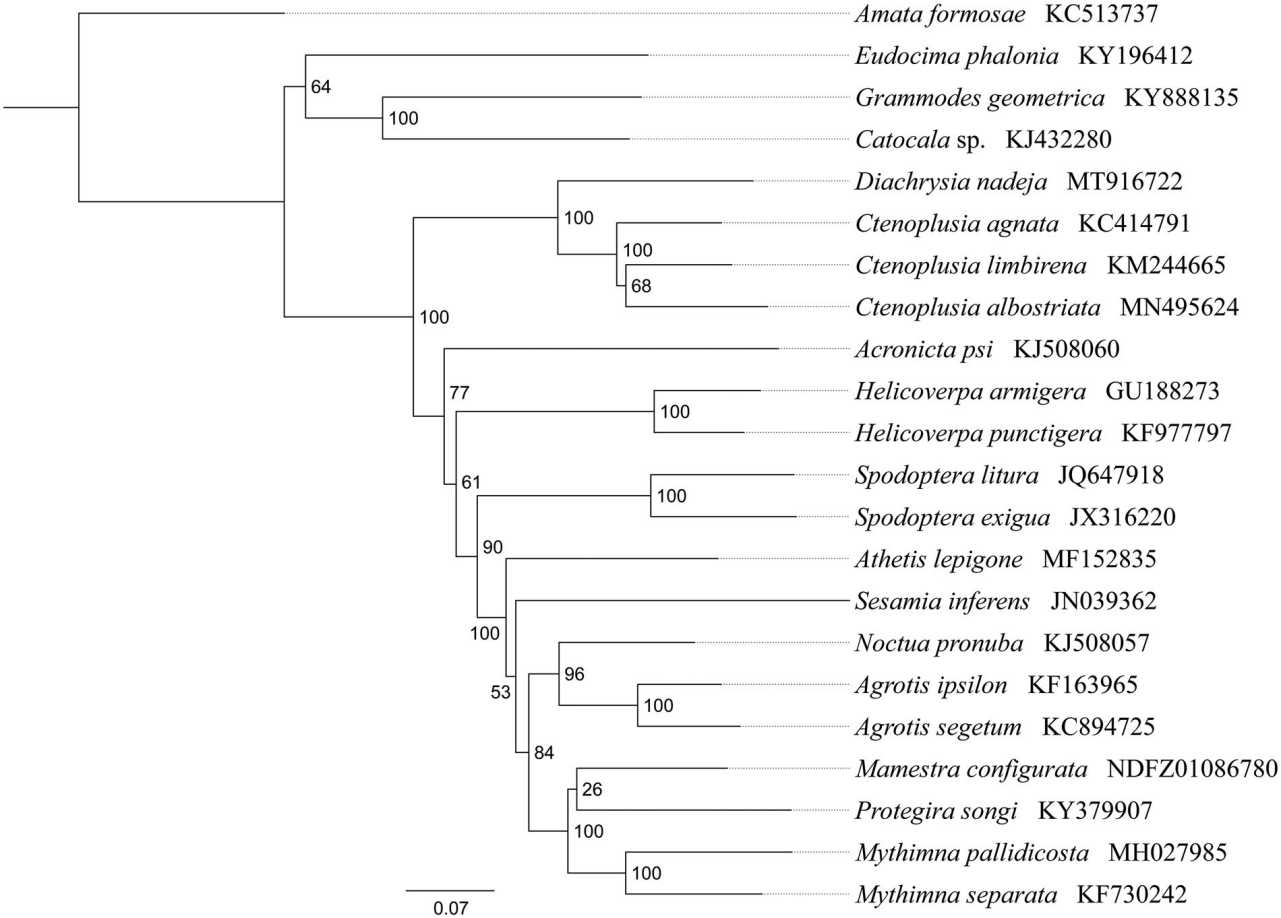
Phylogenetic relationships based on the 13 mitochondrial protein-coding genes sequences inferred from RaxML. Numbers on branches are Bootstrap support values (BS).

## Data Availability

The data that support the findings of this study are openly available in NCBI (National Center for Biotechnology Information) at https://www.ncbi.nlm.nih.gov/, reference number MT916722.
